# Structurally Durable Bimetallic Alloy Anodes Enabled by Compositional Gradients

**DOI:** 10.1002/advs.202201209

**Published:** 2022-04-01

**Authors:** Zhenzhu Wang, Jie Wang, Jiangfeng Ni, Liang Li

**Affiliations:** ^1^ School of Physical Science and Technology Center for Energy Conversion Materials & Physics (CECMP) Jiangsu Key Laboratory of Thin Films Soochow University Suzhou 215006 China; ^2^ Light Industry Institute of Electrochemical Power Sources Suzhou 215699 China

**Keywords:** bimetallic alloy, electrochemical performance, gradient electrode, sodium‐ion batteries

## Abstract

Metals such as Sb and Bi are important anode materials for sodium‐ion batteries because they feature a large capacity and low reaction potential. However, the accumulation of stress and strain upon sodium storage leads to the formation of cracks and fractures, resulting in electrode failure upon extended cycling. In this work, the design and construction of Bi_x_Sb_1−x_ bimetallic alloy films with a compositional gradient to mitigate the intrinsic structural instability is reported. In the gradient film, the top is rich in Sb, contributing to the capacity, while the bottom is rich in Bi, helping to reduce the stress in the interphase between the film and the substrate. Significantly, this gradient film affords a high reversible capacity of ≈500 mAh g^−1^ and sustains 82% of the initial capacity after 1000 cycles at 2 C, drastically outperforming the solid‐solution counterpart and many recently reported alloy anodes. Such a gradient design can open up the possibilities to engineering high‐capacity anode materials that are structurally unstable due to the huge volume variation upon energy storage.

## Graphical abstract

The design of structurally durable bimetallic alloy films with a compositional gradient is demonstrated to mitigate the intrinsic structural instability. This gradient film features a high density, a high capacity of ≈500 mAh g^−1^, and sustains 82% of the initial capacity upon 1000 cycles at 2 C, drastically outperforming the solid‐solution counterpart and many alloy anodes.

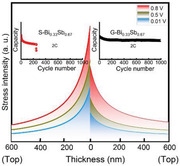

## 1 Introduction

Metals such as Sn, Sb, and Bi are important for sodium‐ion batteries because they are capable of accommodating a large amount of Na ions through a reversible (de)alloying process.^[^ ^1^ ^]^ Among these alloy anodes, Sb is considered a particularly competitive option because it offers a high capacity of 660 or 4400 mA h cm^−3^ (to form Na_3_Sb) at an average potential of 0.5 V versus Na^+^/Na.^[^ ^2^ ^]^ This alloying potential avoids the possibility of sodium plating but does not significantly compromise the working voltage and therefore the energy of full cells. A major challenge lies with the huge volume variation, which could be up to 296% upon the accumulation of Na ions. Such an expansion induces large stress and strain inside electrodes, thus resulting in possible electrode fracture and loss of electrical connection.^[^ ^3^ ^]^ As a result, cycling instability has been commonly reported in Sb anodes.

Given this challenge, two common engineering strategies, i.e., incorporating a carbon matrix and designing a porous structure, have been proposed. Carbon is a common material to buffer the volume swelling of alloying electrodes,^[^ ^4^ ^]^ while the pores provide additional space to accommodate the volume expansion.^[^ ^5^ ^]^ While both designs help to minimize or even diminish the cumulative stress within the Sb electrode, they inevitably lead to a reduction in the volumetric capacity.^[^ ^6^ ^]^ In addition, introducing a less swelling element to construct bimetallic alloys that represents another plausible approach. The introduced element can be either Na inactive or Na active. In the former, inactive components, such as Cu, Ni, Zn, serve as a conductive framework to buffer swelling upon Na addition.^[^ ^7^ ^]^ In the latter scenario, the introduced element is electrochemically active to Na but exhibits a lower degree of volume variation. A representative example is Bi, which undergoes a swelling rate of 223% when Bi is transformed into Na_3_Bi (Table S1, Supporting Information).^[^ ^8^ ^]^ To date, this design has been extensively implemented, as Bi and Sb share a similar structure and are miscible at any proportion in principle.^[^ ^9^ ^]^ For instance, Gao et al. reported a Bi_2_Sb_6_ alloy anode that retained a capacity of 429 mAh g^−1^ over 100 cycles and 258 mAh g^−1^ over 2000 cycles.^[^ ^10^ ^]^ Previously, our group reported a durian‐like Sb_0.25_Bi_0.75_ alloy electrode achieving capacity retention of 284 mAh g^−1^ over 2000 cycles.^[^ ^11^ ^]^ It is noted that a high degree of stability has only been attainable in the high‐Bi‐ratio phases (low capacity) of Bi_x_Sb_1−x_; the high‐Sb‐ratio phases, however, are still plagued by the electrochemical instability caused by the large strain and stress developed.

One plausible way to bypass this dilemma is to engineer a compositional gradient. In this structure, the perfect combination of multiple functional components at the nanoscale offers a possibility to regulate the dissipation of the stress induced by the volume variation.^[^ ^12^ ^]^ Therefore, electrode integrity could be principally preserved regardless of swelling. The conceptual design of gradient structures has been previously pursued in lithium‐ion batteries,^[^ ^13^ ^]^ photoelectrochemical devices,^[^ ^14^ ^]^ and electrocatalysis.^[^ ^15^ ^]^ A case in point is that Sun et al. proposed a compositional gradient structure in the outer layer of Ni‐Mn‐Co oxide particles, which offer high energy and superior thermal stability.^[^ ^16^ ^]^ Recently, a full concentration‐gradient design has been demonstrated, endowing lithium‐rich oxide with excellent voltage retention and rate capability.^[^ ^17^ ^]^ To the best of our knowledge, however, the gradient design has not been well fulfilled in sodium alloy anodes.

In this work, we demonstrate that electrochemical stability can be readily realized in Sb‐rich alloy anodes of compositional gradient Bi_0.33_Sb_0.67_ (G‐Bi_0.33_Sb_0.67_) films without further engineering. This gradient Bi_0.33_Sb_0.67_ film was fabricated by controlled electrodeposition from solutions of varying Bi:Sb ratios. A gradient variation in the concentration of Bi and Sb is presented in the vertical direction from the film bottom to the top. The top is rich in the high‐capacity component of Sb, which contributes to the majority of the capacity, while the bottom is rich in the low‐capacity Bi, which helps to reduce the stress in the interphase between the film and the substrate. Compared with the solid solution of Bi_0.33_Sb_0.67_ (S‐Bi_0.33_Sb_0.67_), the gradient design significantly reduces the stress developed within the electrode film upon sodiation, as confirmed by finite element analysis (FEA). Electrochemical (de)sodiation test results show that G‐Bi_0.33_Sb_0.67_ retains a stable capacity of 328 mAh g^−1^ after 1000 cycles at a rate of 0.5 C (1 C is arbitrarily taken as 500 mA g^−1^). It also displays durable sodium storage at a higher rate of 2 C, thereby demonstrating its great potential in sodium‐ion batteries for renewable and grid storage.

## 2 Results and Discussion

The failure of sodium‐ion batteries is often caused by electrode fracture as a result of the accumulation of stress and strain during electrochemical cycling. Upon the uptake and release of Na^+^ ions, alloy anodes will accordingly experience volume expansion and shrinkage. Once the stress induced by the volume variation surpasses the elastic limit, the electrode structure will undergo permanent deformation and even cracks and fractures. On the one hand, some fractures will lose electrical connection with the current collector, making them inactive in the sequential process. On the other hand, electrode fracturing leads to a fresh surface and continuous generation and growth of the solid‐electrolyte interface (SEI), resulting in augmented resistance and sodium consumption. As this process continues, the failure of electrodes and batteries will eventually occur. Hence, efficient reduction and dissipation of stress are critically desirable while designing durable and sustainable battery electrodes.^[^ ^18^ ^]^

**Figure 1:**
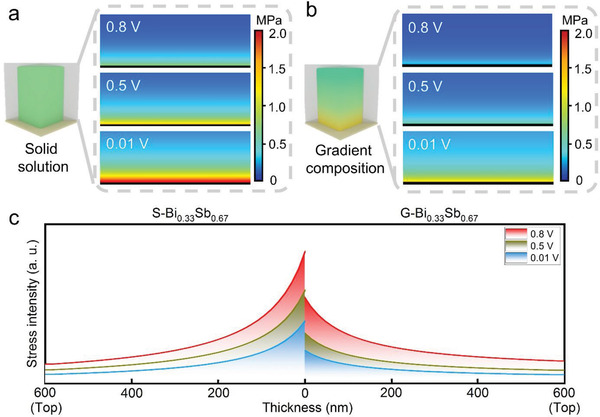
Stress distribution in the solid solution or the gradient structure of bimetallic alloy films simulated by FEA. Stress profiles at different sodiation voltages for a) S‐Bi_0.33_Sb_0.67_ and b) G‐Bi_0.33_Sb_0.67_. c) Comparison of the geometric distribution of stress from the bottom to the top for both samples. The electrode thickness is set at 600 nm.

The design of gradient structures could dissipate the accumulative stress within electrodes. To unveil the effectiveness of such a design, FEA was implemented on varied sodiation stages for both gradient structure and solid solution. The simulated results of G‐Bi_0.33_Sb_0.67_ and S‐Bi_0.33_Sb_0.67_ are presented in **Figure** **1**a,b, respectively. In general, with the storage of Na^+^ ions (sodiation), stress is continuously developed in both electrodes as a direct result of volume swelling. A close observation reveals two distinct features related to stress evolution. First, stress is developed and accumulated preferentially on the electrode bottom. This is because the swelling of the film bottom, not the surface, is primarily restricted by the current collector. Second, the stress gathered in G‐Bi_0.33_Sb_0.67_ is consistently lower than that in S‐Bi_0.33_Sb_0.67_, regardless of the sodiation state. This can be explained by the unique composition gradient where less‐swelling Bi is enriched at the bottom of G‐Bi_0.33_Sb_0.67_. Figure 1c illustrates the cross‐sectional stress distribution in both electrodes. The maximal stress arising in G‐Bi_0.33_Sb_0.67_ is 35% less than that in S‐Bi_0.33_Sb_0.67_ at the same sodiation stages (Figure S1, Supporting Information). Remarkably, the accumulation of stress near the bottom is much slower in G‐Bi_0.33_Sb_0.67_ versus in S‐Bi_0.33_Sb_0.67_, thus more effectively preventing the former from possible cracking and fracturing. Therefore, designing a compositional gradient could theoretically address the instability issue relative to bimetallic alloy anodes.

Guided by the simulation results, we constructed gradient films of G‐Bi_0.33_Sb_0.67_ by galvanostatic electrodeposition. As schematically illustrated in Figure S2 (Supporting Information), a compositional gradient was realized involving varying Bi:Sb ratios in the electrolyte. The initial electrolyte consists of 50 mM Bi^3+^ and Sb^3+^. With the processing of deposition, a 50 mM solution of Sb^3+^ was continuously poured into the electrolyte, leading to gradual dilution of Bi^3+^ species. The concentration of Sb remains unchanged because of the supply of Sb^3+^ cations. Such a variation in the Bi:Sb ratio leads to the generation of compositional gradients. Scanning electron microscopy (SEM) images reveal a dense film deposited on Cu foil with a rough surface for the as‐deposited films (Figure S3, Supporting Information). The film, ≈630 nm in thickness, contains 33% Bi and 67% Sb as a whole, as determined by inductively coupled plasma atomic emission spectrometry. As a comparison, solid‐solution films were deposited from an electrolyte of 25 × 10^−3^ m Bi^3+^ and 50× 10^−3^ m Sb^3+^.

**Figure 2:**
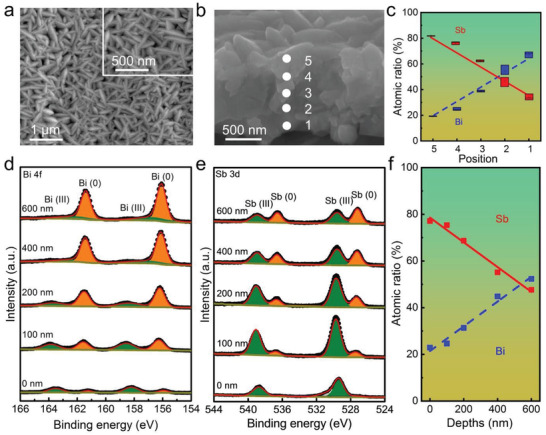
Morphology and compositional gradient of G‐Bi_0.33_Sb_0.67_. a) Top‐view and b) cross‐sectional SEM images. The numbers in the cross section indicate the sites where the EDS signals were recorded. c) Atomic ratios of Bi and Sb showing the compositional gradient. Data acquisition by EDS was repeated three times. Core‐level XPS spectra of d) Bi 4f and e) Sb 3d recorded at different depths by Ar^+^ ion etching. f) Atomic ratio of Bi and Sb by XPS, again showing the compositional gradient.

After mild annealing at 200 °C, the gradient film retains its original morphology (**Figure** **2**a). To verify the gradient structure, we conducted compositional analysis by energy‐dispersive X‐ray spectroscopy (EDS). The composition was recorded at equidistant positions in the cross‐section of G‐Bi_0.33_Sb_0.67_ (Figure 2b,c). The results present a vertical gradient distribution of Bi and Sb from the film bottom to the top. The bottom is rich in Bi, and the top is rich in Sb. Both elements exhibit a gradient and reciprocal transition in the film. This gradient is closely linked with the variation in ion concentrations and electrode potentials during deposition. In the initial stage, the phase deposited from the solution of 50 mM Bi and Sb is dominated by Bi because the standard electrode potential of Bi^3+^/Bi (0.32 V vs SHE) is more positive than that of Sb^3+^/Sb (0.21 V vs SHE).^[^ ^11^ ^]^ With the processing of deposition, the dilution of electrolyte Bi species results in a gradual shift of the redox potential of Bi^3+^/Bi toward lower values. In contrast, the electrolyte concentration of Sb remains unchanged because of the continuous replenishment of the Sb^3+^ solution. When the redox of Bi^3+^/Bi is more negative than that of Sb^3+^/Sb in the electrolyte, the deposition of Sb species would be dominant. As a result, a vertical gradient structure of Bi_x_Sb_1−x_ is created. If no solution of Sb^3+^ flows into the electrolyte, both Bi and Sb will be consumed, and a solid solution of Bi_x_Sb_1−x_ will be deposited. As seen in Figure S4, Supporting Information, the film deposited from an electrolyte of 25× 10^−3^ m Bi and 50× 10^−3^ m Sb^3+^ discloses a solid solution with a ratio of 33% Bi and 67% Sb. In addition, the atomic composition of G‐Bi_0.33_Sb_0.67_ films with depth was inspected by X‐ray photoelectron spectroscopy (XPS) combined with Ar^+^ ion etching. As presented in Figure 2d–f, the signal intensity of Sb gradually decreases with etching, while that of Bi correspondingly increases. Again, the compositional gradient is detected for both Bi and Sb, thus validating the construction of gradient films of Bi_x_Sb_1−x_.

**Figure 3:**
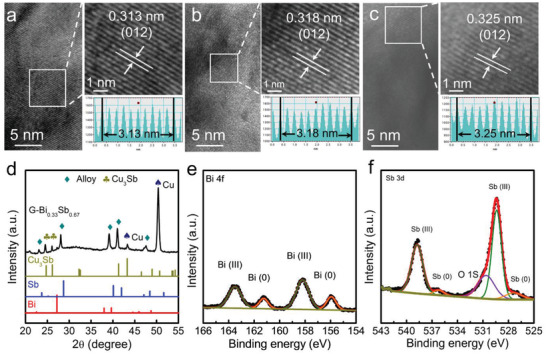
Structural analysis of G‐Bi_0.33_Sb_0.67_. a–c) High‐resolution TEM images showing *d*‐spacings of a) 0.313 nm, b) 0.318 nm and c) 0.325 nm, correlating to the (012) plane of the hexagonal alloy phase with varying Bi:Sb ratios. d) XRD pattern. Core‐level XPS spectra of e) Bi 4f and f) Sb 3d.

The gradient structure was further corroborated by high‐resolution transmission electron microscopy (TEM) images observed at distinct sites of the film. The TEM images in **Figure** **3**a–c reveal a good crystalline state with lattice fringes corresponding to the (012) plane of Bi_x_Sb_1−x_ alloys.^[^ ^19^ ^]^ The interplanar spacings show some variation, ranging from 0.313 (Figure 3a) to 0.318 nm (Figure 3b) and 0.325 nm (Figure 3c). As the spacings are in the midst of those of Sb (0.310 nm) and Bi (0.328 nm), this variation could be used to support the composition gradation in the film. In the X‐ray diffraction (XRD) pattern in Figure 3d, the G‐Bi_0.33_Sb_0.67_ film can be indexed into a hexagonal structure with the space group of R$\bar{3}$m. Two additional diffraction peaks at 24.7° and 26.1° can be assigned to Cu_3_Sb (PDF #11‐0072), which functions as a metal glue to consolidate the film and the substrate.^[^ ^20^ ^]^ In addition, XPS was adopted to probe the surface state of the gradient film (Figure 3e,f). Both metal elements exhibit two sets of doublets, which can be attributed to trivalent and metallic Bi (or Sb), respectively. The appearance of Bi^3+^ and Sb^3+^ is a direct result of the exposure of the film in the ambient atmosphere, as corroborated by the abnormal signal intensity of O 1s in Figure 3f.^[^ ^21^ ^]^ Interestingly, the portion of Sb^3+^ in Sb species is overwhelmingly greater than that of Bi^3+^, suggesting that the former is more sensitive to oxidation.

**Figure 4:**
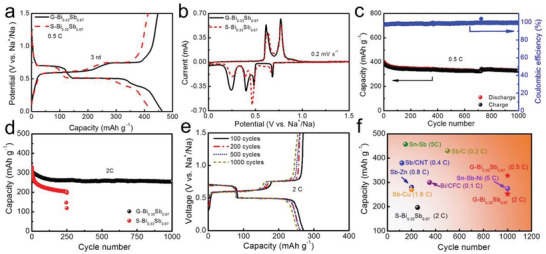
Electrochemical performance of bimetallic alloy films for Na^+^ storage. Comparison of a) typical GCD and b) CV curves of both films_._ c) Electrochemical cycling of G‐Bi_0.33_Sb_0.67_ at a rate of 0.5 C. d) Comparison of electrochemical cycling of G‐Bi_0.33_Sb_0.67_ and S‐Bi_0.33_Sb_0.67_ at a rate of 2 C. The S‐Bi_0.33_Sb_0.67_ cell undergoes a capacity plummet after 250 cycles. Prior to the test, the cells were activated for three cycles at 0.5 C. e) GCD curves of G‐Bi_0.33_Sb_0.67_ at different cycles. f) Electrochemical stability of G‐Bi_0.33_Sb_0.67_ in comparison with recently published alloy anodes.

To unravel the superiority of the gradient structure, sodium storage of both Bi_0.33_Sb_0.67_ films was electrochemically evaluated by galvanostatic charge and discharge (GCD) and cyclic voltammetry (CV). **Figure** **4**a compares the GCD curves of Na half cells in the third cycle. G‐Bi_0.33_Sb_0.67_ affords a higher reversible capacity of 447 mAh g^−1^ versus S‐Bi_0.33_Sb_0.67_, which only affords 412 mAh g^−1^. Remarkably, both films present a high sodium activity in the initial stage, exhibiting a high desodiation capacity of up to ≈500 mAh g^−1^ (Figure S5, Supporting Information). However, the solid solution undergoes a more significant irreversible loss and capacity decay during the initial cycling, possibly caused by the larger stress and strain. In the CV profile shown in Figure 4b, three cathodic peaks located at 0.48, 0.39, and 0.24 V represent stepwise alloying with Na.^[^ ^22^ ^]^ The extra peak at 0.67 V might be attributed to the formation of an SEI, which causes initial capacity loss. By analogy, desodiation in Bi_x_Sb_1−x_ films proceeds stepwise. The two anodic peaks at 0.61 and 0.77 V reflect the two‐step dealloying of Na_3_Bi_x_Sb_1−x_ to Na(Bi, Sb) and Bi_x_Sb_1−x_, in good agreement with previous reports.^[^ ^10^, ^11^ ^]^

Previous studies have revealed that Sb and Sb‐rich alloys suffer from poor structural integrity due to volume swelling and cumulative stress on sodium cycling.^[^ ^11^ ^]^ By designing a compositional gradient, the swelling and strain could be redistributed, and their accumulation could be dissipated. This assumption has been well manifested by the superior cycling stability of G‐Bi_0.33_Sb_0.67_, as shown in Figure 4c–e. The gradient film affords 328 mAh g^−1^ after 1000 cycles at a rate of 0.5 C, and 253 mAh g^−1^ over 1000 cycles at a high rate of 2 C. The capacity decay per cycle is only 0.018%. Electrode durability at higher rates (such as 5 C) and extended cycles (2000 cycles) is also attainable (Figure S6, Supporting Information). In contrast, the solid‐solution counterpart only sustains ≈250 cycles, after which it experiences a capacity plummet. Moreover, G‐Bi_0.33_Sb_0.67_ shows reproducible GCD curves at various cycling stages, highlighting the acquisition of electrode stability through such a unique gradient design. Remarkably, this gradient film electrode is fully comparable or even outperform some nanoscale Bi and Sb anodes (Figure 4f).^[^ ^23^ ^]^ Also, our dense film electrodes feature a much higher packing density (6.8 g cm^−3^) versus those of composite electrodes,^[^ ^24^ ^]^ which is essential and unique for microbatteries to power microelectronics.^[^ ^25^ ^]^

**Figure 5:**
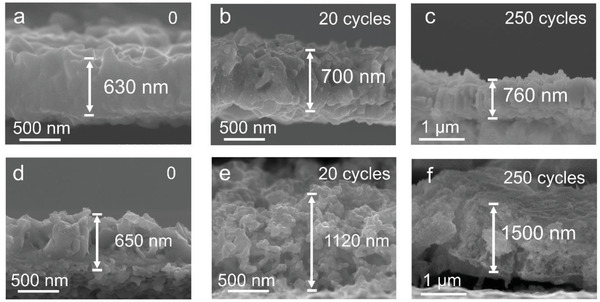
Comparison of electrode evolution upon electrochemical cycling. Cross‐sectional SEM images of a–c) G‐Bi_0.33_Sb_0.67_ and d–f) S‐Bi_0.33_Sb_0.67_ after 0, 20, and 250 cycles, respectively.

To monitor the evolution of electrodes upon sodium cycling, cross‐sectional SEM imaging was conducted on the electrode at various cycling stages. While G‐Bi_0.33_Sb_0.67_ almost retains the original state regardless of cycling (**Figure** **5**a–c), S‐Bi_0.33_Sb_0.67_ reveals substantial evolution in the film thickness and morphology (Figure 5d–f). The augmentation of the thickness of S‐Bi_0.33_Sb_0.67_ is accompanied by the generation of pores and fractures. Delamination of the film from the substrate is also visible, which is believed to be responsible for the sudden capacity drop observed in Figure 4d. This result indicates that the gradient structure could effectively dissipate the stress and thus avoid possible electrode failure. In addition, XRD analysis reveals that no evident structural change occurs, even after 250 continuous cycles (Figure S7, Supporting Information). Furthermore, electrochemical impedance spectroscopy confirms the electrode durability during cycling (Figure S8, Supporting Information). While the impedance spectrum of G‐Bi_0.33_Sb_0.67_ shows little evolution, that of S‐Bi_0.33_Sb_0.67_ constantly evolves with cycling. The marked evolution of charge‐transfer resistance (*R*_ct_) could be linked with the formation of pores and fractures that facilitate the permeation of electrolyte ions in S‐Bi_0.33_Sb_0.67_. In addition, the *R*_ct_ of G‐Bi_0.33_Sb_0.67_ is consistently less than that of S‐Bi_0.33_Sb_0.67_, implying that the former is more efficient for the charge transfer reaction.

**Figure 6:**
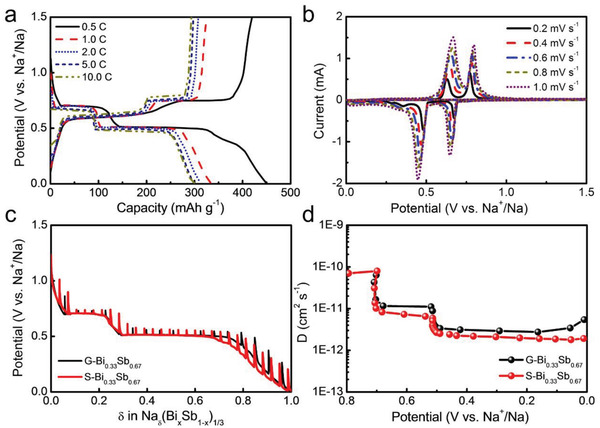
Kinetic analysis of Bi_0.33_Sb_0.67_ film electrodes upon sodium‐ion storage. a) GCD and b) CV curves of G‐Bi_0.33_Sb_0.67_ at varying rates. c) GITT curves of G‐Bi_0.33_Sb_0.67_ and S‐Bi_0.33_Sb_0.67_. During the test, the cell was sodiated at 0.2 C for 600 s, followed by a relaxation of 1800 s. d) Na^+^ diffusion coefficients of G‐Bi_0.33_Sb_0.67_ and S‐Bi_0.33_Sb_0.67_ as a function of potential.

Significantly, the gradient film also features robust kinetic behavior, as shown in **Figure** **6**. At current rates of 0.5, 1, 2, 5, and 10 C, G‐Bi_0.33_Sb_0.67_ affords reversible capacities of 413, 353, 337, 327, and 319 mAh g^−1^, respectively, which are much greater than those of S‐Bi_0.33_Sb_0.67_ (Figure S9, Supporting Information). Notably, the charge and discharge profiles almost overlap regardless of the current rates, suggesting that the polarization due to Ohmic resistance and ion diffusion is negligible.^[^ ^26^ ^]^ Similarly, CV curves of G‐Bi_0.33_Sb_0.67_ at various scan rates ranging from 0.2 to 1 mV s^−1^ reveal an almost constant peak position, again reflecting a high degree of reversibility and robustness. To quantitatively probe the disparity between these two samples, we further measured the apparent diffusion coefficient of Na ions (*D*_Na_) in the films by the galvanostatic intermittent titration technique (GITT) according to the following equation:^[^ ^27^ ^]^

(1) $$D\;=\frac{4}{\pi }\;{\left(\frac{I_{0}V_{m}}{FS}\right)}^{2}{\left(\frac{dE/dx}{dE/d\sqrt{t}}\right)}^{2}$$

where *I*_0_ is the applied current, *V*_m_ is the molar volume, *F* is Faraday's constant, *S* is the surface area, *d* *E*/*d* *x* is the slope of the titration curve at each composition *x*, and *t* is the time. The GITT curves upon sodiation and calculated *D*_Na_ for G‐Bi_0.33_Sb_0.67_ and S‐Bi_0.33_Sb_0.67_ are compared in Figure 6c,d, respectively. For both samples, the diffusion coefficient is in the range of 10^−12^–10^−10^ cm^2^ s^−1^, which is close to the values reported for alloy anodes.^[^ ^28^ ^]^ Importantly, the *D*_Na_ of G‐Bi_0.33_Sb_0.67_ is consistently larger than that of S‐Bi_0.33_Sb_0.67_, and this scenario is more notable near the end of sodiation due to the accumulation of strain. At sodiation of 0.01 V, G‐Bi_0.33_Sb_0.67_ gives a *D*_Na_ value of 5.44 × 1 0^−12^ cm^2^ s^−1^, almost threefold that of S‐Bi_0.33_Sb_0.67_ (1.90 × 10^−12^ cm^2^ s^−1^). First‐principles calculations have predicted that a strain gradient would substantially impede the diffusion of alkali ions because of increased diffusion barriers.^[^ ^29^ ^]^ In the neat design of gradient structures, the strain accumulated inside the film can be efficiently dissipated, and thus, its effect is on the diffusion of Na^+^ could be minimized.

## 3 Conclusion

In summary, we have proposed a gradient structure of bimetallic Bi_x_Sb_1−x_ alloys to mitigate the structural instability upon (de)sodiation. In the gradient structure, the film top is rich in Sb, contributing to the capacity, while the bottom is rich in Bi, helping to reduce the stress developed in the interphase between the film and the substrate. Significantly, the gradient film affords a capacity of ≈500 mAh g^−1^ and sustains 82% of the initial capacity over 1000 cycles at 2 C, drastically outperforming the solid‐solution counterpart and many reported alloy anodes. Such superior stability could be correlated to the specific material design where the accumulation of the stress inside the film could be essentially suppressed and dissipated. The gradient film also exhibits excellent rate capability, affording 319 mAh g^−1^ at a high rate of 10 C. Significantly, such films feature a high packing density of 6.8 g cm^−3^, which is essential and unique for microbatteries to power microelectronics. We believe that the gradient design could open up a broad avenue to engineering high‐capacity anode materials that are plagued by the poor structural integrity caused by the huge volume variation upon energy storage.

## 4 Experimental Section

### Synthesis of Bi_0.33_Sb_0.67_ Films

G‐Bi_0.33_Sb_0.67_ film electrodes were directly grown on Cu foil through galvanostatic electrodeposition. Prior to deposition, Cu foil (0.1 mm, 99%) was ultrasonically cleaned in oxalic acid (1 m), deionized water, and ethanol, respectively. Two electrolytes were prepared for electrodeposition. Electrolyte **A** consists of BiCl_3_ (50 × 10^−3^ m), SbCl_3_ (50 × 10^−3^ m), H_3_BO_3_ (60 × 10^−3^ m), and C_2_H_10_Cl_2_N_2_ (240 × 10^−3^ m) dissolved in a mixture solvent of ultrapure water (20 mL), ethanol (30 mL), ethylene glycol (50 mL), and HCl (3 mL). Electrolyte **B** has a similar composition without BiCl_3_. Electrodeposition was carried out in a three‐electrode setup in electrolyte **A** with Ag/AgCl as the reference electrode and platinum gauze as the counter electrode.^[^ ^11^ ^]^ During deposition, electrolyte **B** was continuously fed into electrolyte **A**. The gradual dilution of Bi^3+^ cations in the electrolyte led to the formation of a compositional gradient. G‐Bi_0.33_Sb_0.67_ films were electrodeposited at a current of 5.7 mA cm^−2^ for 200 s with constant stirring. The mass loading of the film is 0.43 mg cm^−2^. As a comparison, S‐Bi_0.33_Sb_0.67_ films were electrodeposited on Cu foil from an electrolyte of 25 × 10^−3^ m Bi^3+^ and 50 × 10^−3^ m Sb^3+^. The resulting films were thoroughly washed with water and ethanol, dried, and finally annealed at 200 °C for 60 min in a tube furnace under argon flow.

## Conflict of Interest

The authors declare no conflict of interest.

## Supporting information

Supporting Information

## Data Availability

The data that support the findings of this study are available from the corresponding author upon reasonable request.
