# Supplementary material for: Structurally Durable Bimetallic Alloy Anodes Enabled by Compositional Gradients
Source: Adv Sci (Weinh). 2022 Apr 1;9(16):2201209. doi: 10.1002/advs.202201209 (PMC9165509; doi:10.1002/advs.202201209)
Supplement: Supplementary file 1 — Supporting Information [file ADVS-9-2201209-s001.pdf]

ADVANCED SCIENCE
Open Access

Supporting Information

for *Adv. Sci.*, DOI 10.1002/advs.202201209

Structurally Durable Bimetallic Alloy Anodes Enabled by Compositional Gradients

Zhenzhu Wang, Jie Wang, Jiangfeng Ni and Liang Li**

© 2022 Wiley-VCH GmbH

Supporting Information

Structurally Durable Bimetallic Alloy Anodes Enabled by Compositional Gradients

Zhenzhu Wang, Jie Wang, Jiangfeng Ni, and Liang Li**

Z. Wang, J. Wang, Prof. J. Ni, Prof. L. Li,

School of Physical Science and Technology, Center for Energy Conversion Materials & Physics (CECMP), Jiangsu Key Laboratory of Thin Films, Soochow University, Suzhou 215006, China

Prof. J. Ni

Light Industry Institute of Electrochemical Power Sources, Suzhou 215699, China

Email: jeffni@suda.edu.cn, lli@suda.edu.cn

Methods

Materials characterization: As-prepared films were observed by field-emission scanning electron microscopy (Hitachi SU8010) and transmission electron microscopy (FEI Tecnai G2 F20). For the observation of cycled films, the cell was dismantled, and the film was thoroughly washed with dimethyl carbonate to remove electrolyte and preserved in an Ar-filled glove box. The crystalline structure of the samples was identified by X-ray diffraction (Bruker D8 Advance). The chemical composition and states were detected by X-ray photoelectron spectroscopy (ESCALAB 250Xi). The core-level binding energy was corrected with the C 1s binding energy of 284.8 eV.

Finite element analysis: Finite element analysis is a numerical technique used to find approximate solutions to boundary value problems of partial differential equations.[1] This analysis was carried out based on the model proposed by Sastry's group.[2] The diffusion of sodium in the electrode is proportional to the gradient of the sodium concentration profile compromised by the elastic energy of the system. In the mass conservation equation:

$$\frac{\partial c}{\partial t} + \nabla \cdot J = 0 \text{ and } J = -D(\nabla c - \frac{\Omega c}{RT} \nabla \sigma_{\mathrm{h}})$$

where c is sodium ion concentration, D is sodium ion diffusion coefficient, Ω is partial molar volume, R is gas constant, T is temperature, and σ_{h} is hydrostatic stress, defined as $(\sigma_{11}+ \sigma_{22} +\sigma_{33})/3$, where σ_{ij} are the stress tensor elements. The equations above can be solved using the Neumann boundary condition:

$$J \cdot \vec{n} = i/F$$

where $\vec{n}$ is the surface normal vector, i is the electric current density, and F is Faraday's constant. The current density i was selected to be low enough to ensure that the sodium ion concentration was uniform throughout the entire porous structure after Na modification. The stress and strain caused by the insertion of sodium can be expressed as:

$$\varepsilon_{\mathrm{ij}} = \frac{1}{E}\left[(1+\upsilon)\sigma_{\mathrm{ij}} - \upsilon\sigma_{\mathrm{ij}}\delta_{\mathrm{ij}}\right] + \frac{c\Omega}{3}\delta_{\mathrm{ij}}$$

where $\varepsilon_{\mathrm{ij}}$ is the strain component, σ_{ij} is the stress component, and δ_{ij} is the Kronecker delta. A periodic boundary condition was applied on two sides of the block. Data on mechanical characteristics[3] and electrochemical parameters[4] were directly adapted for the simulation.

Electrochemical evaluation: Electrochemical evaluation was performed on 2032-type coin cells. A gradient or solid-solution $Bi_{0.33}Sb_{0.67}$ film with an area of 0.35 cm^2 was directly used as the working electrode. The counter electrode was Na foil, the electrolyte was 1 M $NaPF_6$ dissolved in diglyme, and the separator was a glass microfiber filter (Whatman). Half of the cells were assembled in an Ar-filled glovebox (Mikrouna). Electrochemical sodium storage tests were performed on a LAND battery test system at room temperature. Cyclic voltammetry and electrochemical impedance spectroscopy were measured on an RST electrochemical workstation. The impedance was collected in the frequency range of 100 kHz to 0.1 Hz with an oscillation potential of 7 mV.

Table

Table S1. Volume variations of Bi and Sb during sodiation.

Sample	Density (g cm^{-3})	M_{weight} (g mol^{-1})	M_{volume} (cm^3 mol^{-1})	Product	Density (g cm^{-3})	M_{weight} (g mol^{-1})	M_{volume} (cm^3 mol^{-1})	ΔV%
Bi	9.8	208.98	21.32	Na_3Bi	4.04	277.95	68.80	223%
Sb	6.7	121.76	18.17	Na_3Sb	2.65	190.73	71.97	296%

Figures

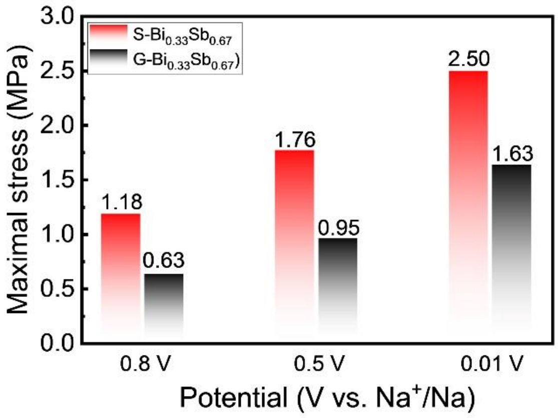

Figure S1. FEA simulation of stress accumulation. Comparison of the maximal stress accumulated at the bottom of G-$Bi_{0.33}Sb_{0.67}$ and S-$Bi_{0.33}Sb_{0.67}$ at different sodiation voltages. The gradient structure could reduce the maximal stress by ~35% in the film bottom.

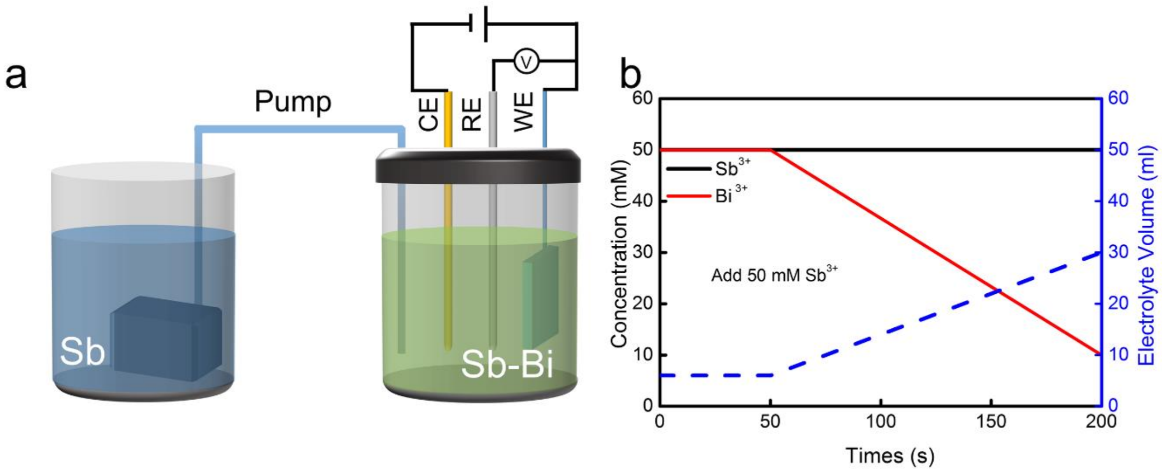

Figure S2. Electrodeposition of G-$Bi_{0.33}Sb_{0.67}$. a) Schematic of the deposition setup. b) Variation in the salt concentration in the electrolyte during deposition. Note that the influence of deposition on the electrolyte cation concentrations is negligible. The initial electrolyte consisted of 50 mM Bi^{3+} and Sb^{3+}. With the processing of deposition, a 50 mM solution of Sb^{3+} was continuously poured into the electrolyte, leading to gradual dilution of Bi^{3+} species.

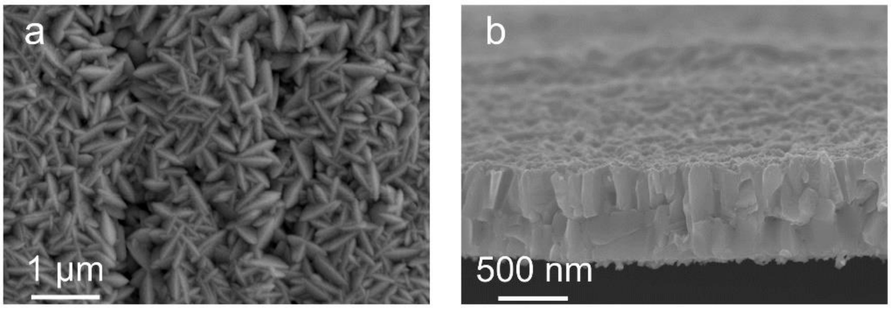

Figure S3. a) Top-view and b) cross-sectional view SEM images of as-deposited G-$Bi_{0.33}Sb_{0.67}$ films. The film thickness is about 630 nm.

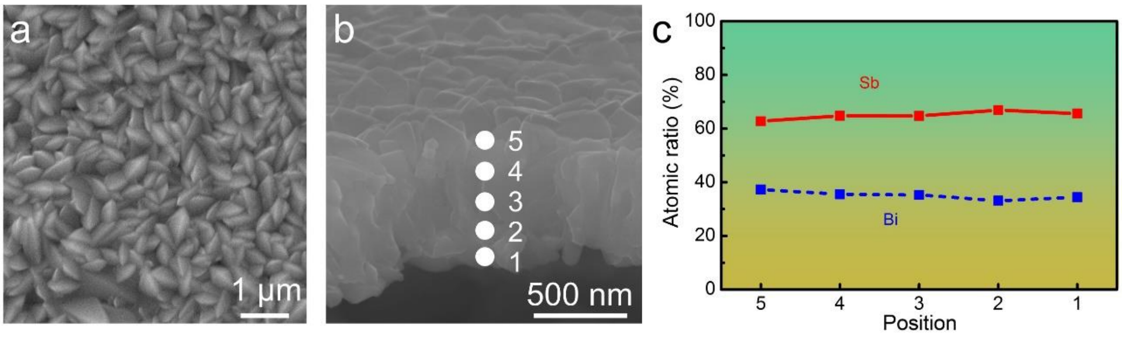

Figure S4. Morphology and compositional variation of S-$Bi_{0.33}Sb_{0.67}$. a) Top-view and b) cross-sectional SEM images. The numbers in the cross-section indicate the sites where the EDS signals were recorded. c) Atomic ratios of Bi and Sb by EDS showing almost constant ratios of Bi and Sb regardless of the positions.

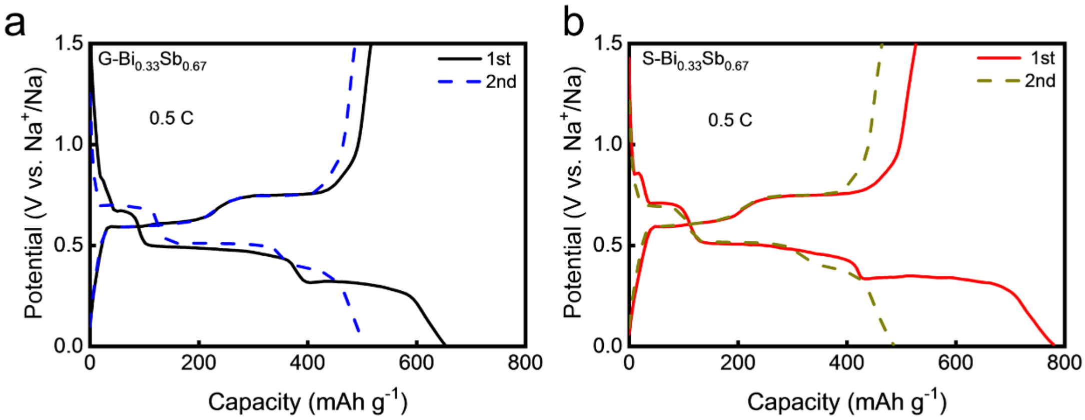

Figure S5. Initial GCD curves of a) G-$Bi_{0.33}Sb_{0.67}$ and b) S-$Bi_{0.33}Sb_{0.67}$ at 0.5 C. Both samples exhibit an initial desodiation capacity of up to 500 mAh g^{-1}. However, G-$Bi_{0.33}Sb_{0.67}$ discloses a more stable behavior by retaining 486 mAh g^{-1} in the second cycle, in comparison to 464 mAh g^{-1} for S-$Bi_{0.33}Sb_{0.67}$.

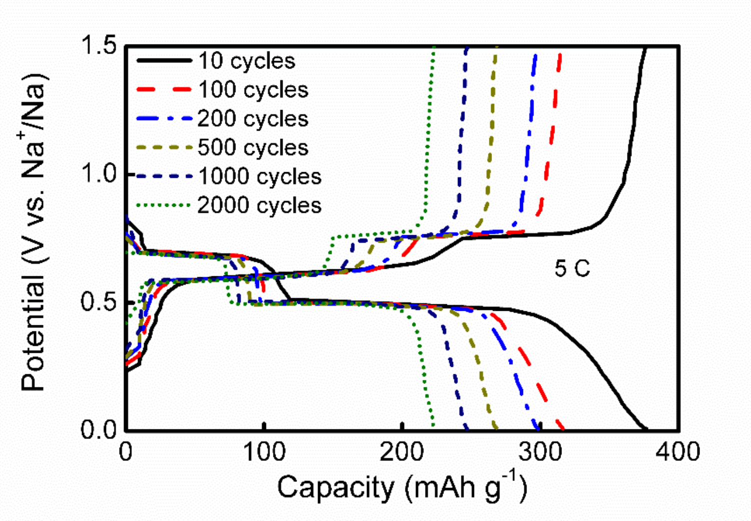

Figure S6. GCD curves of G-$Bi_{0.33}Sb_{0.67}$ at various cycling stages at a high rate of 5 C. After 2000 cycles, a capacity of 223 mAh g^{-1} is retained.

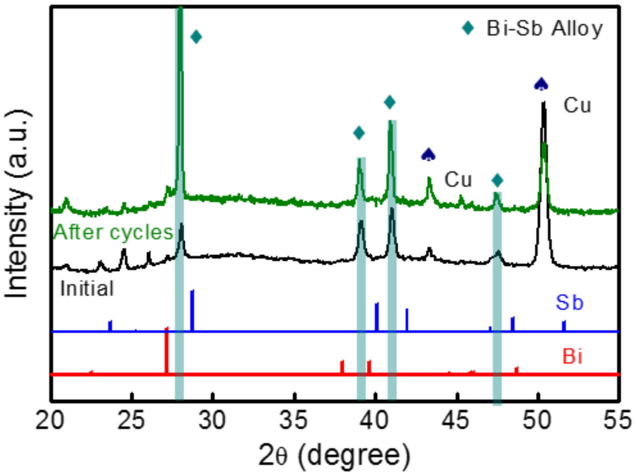

Figure S7. XRD patterns of G-$Bi_{0.33}Sb_{0.67}$ at the initial state and after 250 cycles. No evident structural change is observable.

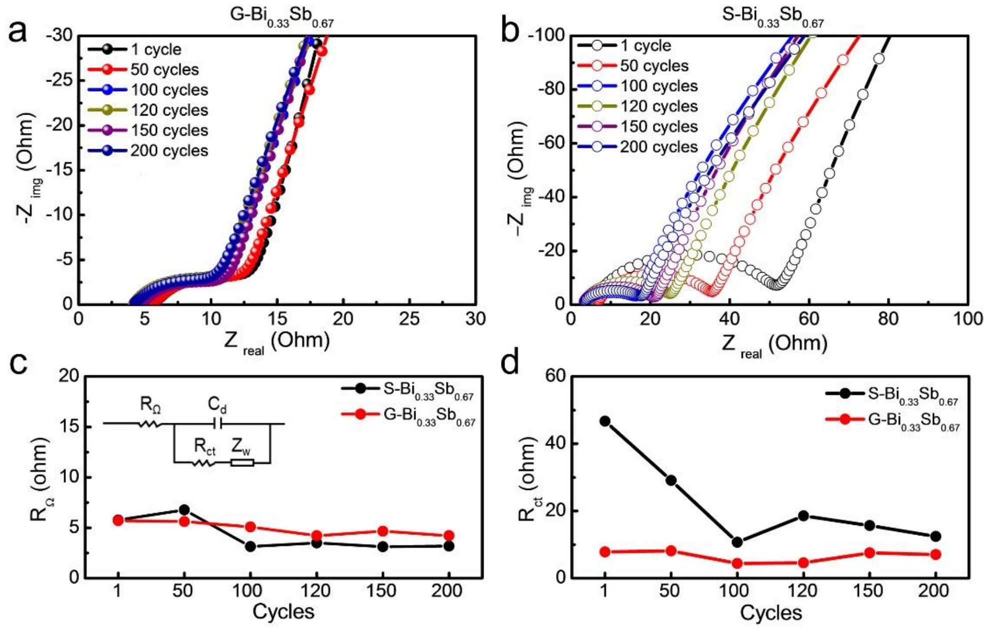

Figure S8. Evolution of EIS results upon cycling. Nyquist plots of a) G-$Bi_{0.33}Sb_{0.67}$ and b) S-$Bi_{0.33}Sb_{0.67}$. Fitted impedance parameters of c) R_{Ω} and d) R_{ct}. The R_{ct} of G-$Bi_{0.33}Sb_{0.67}$ remains unchanged, while the R_{ct} of S-$Bi_{0.33}Sb_{0.67}$ drastically decreases as a possible result of the generation of pores and cracks.

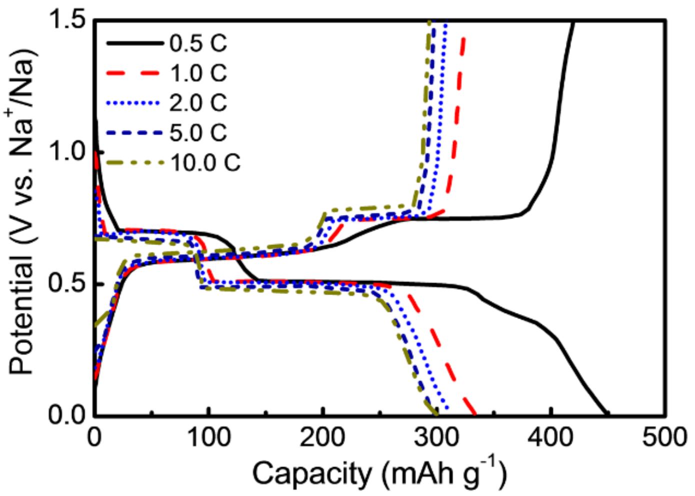

Figure S9. GCD curves of S-$Bi_{0.33}Sb_{0.67}$ at varying current rates. S-$Bi_{0.33}Sb_{0.67}$ affords reversible capacities of 419, 325, 309, 297, and 293 mAh g^{-1} at current rates of 0.5, 1, 2, 5, and 10 C, respectively.

References

[1] a) Z. Wang, J. Ni, L. Li, J. Lu, *Cell Rep. Phys. Sci.* **2020**, *1*, 100078; b) V. Zadin, D. Brandell, H. Kasemägi, A. Aabloo, J. O. Thomas, *Solid State Ionics* **2011**, *192*, 279.

[2] a) X. Zhang, W. Shyy, A. M. Sastry, *J. Electrochem. Soc.* **2007**, *154*, A910; b) J. Park, W. Lu, A. M. Sastry, *J. Electrochem. Soc.* **2011**, *158*, A201.

[3] M. Mortazavi, Q. Ye, N. Birbilis, N. V. Medhekar, *J. Power Sources* **2015**, *285*, 29.

[4] J. H. Park, Y. S. Choi, Y. W. Byeon, J. P. Ahn, J. C. Lee, *Nano Energy* **2019**, *65*, 104041.
